# Resistin Expression Is Associated With Interstitial Lung Disease in Dermatomyositis

**DOI:** 10.3389/fmed.2022.903887

**Published:** 2022-05-03

**Authors:** Lifang Ye, Yu Zuo, Fang Chen, Yuetong Xu, Puli Zhang, Hongxia Yang, Sang Lin, Qinglin Peng, Guochun Wang, Xiaoming Shu

**Affiliations:** ^1^Department of Rheumatology, Key Laboratory of Myositis, China–Japan Friendship Hospital, Beijing, China; ^2^Graduate School of Peking Union Medical College, Chinese Academy of Medical Sciences, Peking Union Medical College, Beijing, China; ^3^Peking University China-Japan Friendship School of Clinical Medicine, Beijing, China

**Keywords:** resistin, interstitial lung disease, dermatomyositis, disease activity, severity

## Abstract

**Objective:**

In the current study, we aimed to assess resistin mRNA levels in the peripheral blood mononuclear cells (PBMCs) of dermatomyositis patients with interstitial lung disease (DM-ILD) and their correlation with disease activity.

**Methods:**

We detected resistin mRNA levels in the PBMCs of 37 DM-ILD, 8 DM patients without ILD, and 19 healthy control (HC) subjects by performing quantitative reverse transcription real-time polymerase chain reaction analysis. Associations between resistin expression levels and major clinical manifestations, laboratory examinations, and disease activity were also analyzed. In addition, resistin expression in lung specimens from patients with DM-ILD was examined *via* immunohistochemistry and immunofluorescence.

**Results:**

Resistin mRNA levels in PBMCs were significantly higher in DM-ILD than that in DM patients without ILD and HCs (*p* = 0.043, 0.014, respectively). Among these DM-ILD patients, the resistin levels were significantly elevated in those with rapidly progressive ILD than in those with chronic ILD (*p* = 0.012). The resistin mRNA levels in DM-ILD positively correlated with serum alanine aminotransferase (r = 0.476, *p* = 0.003), aspartate aminotransferase (r = 0.488, *p* = 0.002), lactate dehydrogenase (r = 0.397, *p* = 0.014), C-reactive protein (r = 0.423, *p* = 0.008), ferritin (r = 0.468, *p* = 0.003), carcinoembryonic antigen (r = 0.416, *p* = 0.011), carbohydrate antigen 125 (r = 0.332, *p* = 0.047), interleukin-18 (r = 0.600, *p* < 0.001), and lung visual analog scale values (r = 0.326, *p* = 0.048), but negatively correlated with the diffusing capacity of carbon monoxide (DLco)% (r = −0.447, *p* = 0.041). Immunohistochemical analysis of resistin showed its elevated expression in the macrophages, alveolar epithelial cells, and weak fibrotic lesions from patients with DM-ILD. Immunofluorescence staining confirmed CD68+ macrophages co-express resistin.

**Conclusions:**

Resistin levels were increased in patients with DM-ILD and associated with disease activity and ILD severity. Therefore, resistin may participate in the pathogenesis of DM-ILD and may act as a useful biomarker.

## Introduction

Dermatomyositis (DM) is characterized by muscle weakness and inflammation of the skeletal muscle with progressive involvement in other organs, particularly in the lungs (1). According to previous findings, interstitial lung disease (ILD) is a major complication of DM with a high, ranging from 20 to 78% (2, 3). ILD is also a leading cause of hospitalization in patients with DM, resulting in increased mortality, and is a negative prognostic factor in patients with DM (3). Based on disease progression, two distinct subtypes of ILD have been described in DM, rapidly progressive interstitial lung disease (RP-ILD) and chronic ILD (4). In general, RP-ILD has an aggressive course with a 3-month survival rate of 51.7–72.4% (5, 6). Therefore, early diagnosis and careful monitoring are essential for the effective management of DM patients with ILD (DM-ILD).

Resistin, discovered in mice in 2001 and known as adipose tissue-specific secretory factor (ADSF) or found in inflammatory zone 3 (FIZZ3), is a 12.5 kDa cysteine-rich secreted protein that is involved in inducing insulin resistance (7, 8). Resistin is mainly expressed by peripheral blood mononuclear cells (PBMCs), macrophages, and myeloid cells (9). Resistin is associated with many inflammatory diseases such as rheumatoid arthritis (RA), systemic lupus erythematosus (SLE), and systemic sclerosis (10–12). Previous studies have shown that resistin can act as a pro-inflammatory cytokine. For example, resistin stimulates human macrophage-like cells and PBMCs to promote the production of pro-inflammatory cytokines (i.e., interleukin [IL]-6, IL-1β, and tumor necrosis factor [TNF]-α) (13–15). Resistin may play important roles in regulating inflammation (16). Under pathophysiological conditions, resistin has been observed in the lungs of patients with cystic fibrosis, scleroderma (SSc), and idiopathic pulmonary fibrosis (17–19). These findings suggest that the lungs are a target for resistin signaling. Resistin may be involved in the pathogenesis of ILD, which is characterized by lung injury and subsequent fibrosis (17–19). Resistin may also be a useful biomarker for ILD (11). Furthermore, serum resistin levels are elevated in patients with idiopathic inflammatory myopathy (IIM) and are associated with myositis-specific anti-Jo-1 antibodies, the overall disease activity index, and muscle damage (15). However, the clinical significance of resistin in DM-ILD has not been elucidated.

Therefore, we assessed the association between resistin mRNA levels in PBMCs and clinical variables and detected resistin expression in lung specimens from patients with DM-ILD using immunohistochemistry.

## Materials and Methods

### Patients

Thirty-seven DM-ILD patients and eight DM patients without ILD, who visited the China-Japan Friendship Hospital between August 2016 and September 2020, were included in this study. All patients were diagnosed based on the criteria of Bohan and Peter (20, 21). ILD was diagnosed by high-resolution computed tomography (HRCT) (22). Patients combined with other connective tissue diseases and an age of onset of <18 years were excluded from this study. In addition, 19 sex- and age-matched healthy controls (HCs) from the Physical Examination Center of China-Japan Friendship Hospital were recruited. The patients' clinical and laboratory data (including demographics, main clinical features, laboratory data, and pulmonary function examination) were obtained from the hospital's electronic medical record. Physician global assessment (PGA) was performed to assess the disease activity of patients with DM, which was recorded on a continuous 10 cm visual analog scale (VAS). Myositis disease activity provides a comprehensive score for the whole body, skin, joints, lungs, heart, gastrointestinal system, and muscle organs or systems (23). Eight patients in this study were followed for 3–20 months. Each participant provided written informed consent before enrollment, and the study was approved by the Research Review Committee and Ethics Review Committee of the China-Japan Friendship Hospital.

### Classification of ILD

Patients with DM-ILD were divided into two clinical subsets: those with RP-ILD and those with chronic ILD. RP-ILD is defined as rapidly progressing ILD with severe dyspnea symptoms and new interstitial abnormalities, upon HRCT examination, within 3 months (22). The diagnosis of chronic ILD is according to asymptomatic, slowly progressive ILD or non-RP-ILD imaging for more than 3 months (22).

### Measurement of Resistin

PBMCs were separated from 6 ml peripheral blood samples from patients before immunosuppressive therapy during first hospitalization in patients with DM *via* Histopaque density-gradient centrifugation and stored in liquid nitrogen until the experiments were performed. A Rapid RNA Extraction Kit (Yishan, Shanghai, China) was used to extract total RNA from PBMCs, and NanoDrop 2000 spectrophotometer (Thermo Scientific, USA) was used to quantify the RNA concentrations. PrimeScriptTM RT reagent (Takara Bio Inc., Japan) was performed to reverse-transcribe RNA into complementary DNA. To compare resistin mRNA levels between groups, quantitative reverse transcription real-time polymerase chain reaction analysis was performed using an ABI 7500 sequence detection system (Applied Biosystems, Foupsster City, CA) with SYBR Green Master Mix (Qiagen, Hilden, Germany) and an appropriate forward primer (5′- CTGTTGGTGTCTAGCAAGACC-3′) and reverse primer (5′- CCAATGCTGCTTATTGCCCTAAA-3′). The thermocycling conditions were as follows: 95°C for 2 min, followed by 40 cycles of 95°C for 5 s and 60°C for 30 s. Ribosomal protein S18 (RPS18) mRNA expression was detected as an internal reference for gene-expression analysis. Each sample was measured in triplicate. The 2^−Δ*Ct*^ method was used to calculate the relative RNA expression levels, which were normalized to an endogenous control.

### Immunohistochemistry

Lung biopsy specimens obtained by percutaneous lung biopsies were analyzed using immunohistochemistry. The tissues were fixed with 10% formalin, embedded in paraffin (6 μm thickness), deparaffinized with xylene, and preheated for 30 min in epitope-retrieval solution (Citric Acid Retrieval Solution, Aladdin, Shanghai, China). Then blocked with goat serum for 2 h. The tissue specimens were incubated with rabbit anti-resistin polyclonal antibody (1:200 dilution; Proteintech, Wuhan, China) and anti-CD68 (1:50; Abcam, Cambridge, MA, USA) overnight at 4°C. Goat anti-rabbit IgG secondary antibody (Gene Tech Shanghai Company Limited, Shanghai, China) was used to incubate the tissue specimens for 30 min at 25°C according to the manufacturer's instructions. Peroxidase activity was determined using 3,3′-diaminobenzidine (Gene Tech Shanghai Company Limited). The tissues were then counterstained with hematoxylin and observed under the microscope (Olympus, Tokyo, Japan).

### Immunofluorescence Staining

Lung biopsy specimens were deparaffinized with xylene. Then, citric acid solution (Aladdin, Shanghai, China) was used for antigen retrieval at 100°C for 30 min. 0.3% Triton X-100 was used for cell membrane penetration. The samples were then blocked with goat serum for 2 h. The tissue specimens were then incubated with anti-Resistin (1:200 dilution; Proteintech, Wuhan, China), and anti-CD68 (1:50; Abcam, Cambridge, MA, USA) overnight at 4°C, and then with Alexa 488-conjugated or Alexa 555-conjugated secondary antibody (1:1,000; Cell Signaling Technology, Massachusetts, USA) for 30 min at 25°C. Next, 4′,6-diamidino-2-phenylindole (DAPI; Beyotime) was used to stain the cell nuclei, which were observed under a fluorescence microscope (Olympus, Tokyo, Japan). Image J software was then used for image analysis and merging.

### Measuring Serum IL-18 Levels

During the first visit, blood samples were obtained for IL-18 testing, and the data were compared with routine blood test results. The sera were stored at −80°C until they were determined. Serum IL-18 levels were measured using a Human Total IL-18 Valukine™ ELISA Kit (Novus Biologicals, USA), according to the manufacturer's instructions.

### Statistical Analysis

Normally distributed data were described using the mean ± standard deviation (SD) and were compared by unpaired *t*-test. Non-normally distributed data were described using median [interquartile range (IQR)] and were compared by Mann-Whitney *U*-test. Spearman's correlation analysis was performed to assess correlations. Paired analyses between pre- and post-treatment samples were tested using the Wilcoxon's signed-rank test. Differences at *p* < 0.05 were considered statistically significant. Statistical analysis of the data was analyzed by using GraphPad Prism 8.0 and SPSS 25.0.

## Results

### Clinical Features of the Patients Enrolled in This Study

Thirty-seven patients with DM-ILD were included in the study. Of these, 25 were women. The mean age at onset was 52.89 years, and the median disease duration was 6 months. The disease status was active at the baseline, as shown by laboratory data and physician VAS scores. Clinical manifestations, laboratory test results, and pulmonary function parameters are described in Table 1.

**Table 1:** Characteristics of patients.

**Characteristics**	**Patients (*n* = 37)**
Female/male ratio	25/12
Onset age (years, mean ± SD)	52.89 ± 9.72
Disease duration [months, median (IQR)]	6.0 (2.5–11.5)
Clinical features, no. (%)	
Muscle weakness	14 (37.8%)
Myalgia	13 (35.1%)
Heliotrope rash	19 (51.3%)
Gottron papules/sign	18 (48.6%)
Mechanic's hands	17 (45.9%)
V sign	15 (40.5%)
Skin ulcers	3 (8.1%)
Arthritis/arthralgia	11 (29.7%)
Dysphagia	4 (10.8%)
RP-ILD	12 (32.4%)
Laboratory examinations	
Anti-MDA5, no. (%)	25 (67.5%)
Anti-ARS, no. (%)	5 (13.5%)
Other MSAs, no. (%)	4 (10.8%)
MSA negative, no. (%)	3 (8.1%)
CK, IU/l	95 (32–307)
LDH, IU/l	307.0 (231.5–447.5)
CRP, mg/dl	0.49 (0.25–0.86)
ESR, mm/H	23 (7–40.5)
Ferritin, ng/ml^a^	565.8 (140.7–1,215)
Pulmonary function test	
FVC (%)^b^	82.34 ± 22.13
FEV1 (%)^b^	75.96 ± 18.87
DLCO (%)^b^	65.23 ± 17.64
Physician VAS	5.0 ± 2.4

*ARS, aminoacyl-tRNA synthetases; CK, creatine kinase; CRP, C-reactive protein; DLCO, percent carbon monoxide diffusion capacity; DM, dermatomyositis; ESR, erythrocyte sedimentation rate; FEV1, predicted forced expiratory volume in 1 s; FVC, predicted forced vital capacity; ILD, interstitial lung disease; LDH, lactic dehydrogenase; MDA-5, melanoma differentiation-associated gene-5; MMT8, manual muscle testing of eight muscle groups; MSA, myositis-specific antibodies; VAS, visual analog scale. ^a, b^The data shown represent 36 and 34 patients, respectively*.

### Expression of Resistin MRNA From PBMCs in Patients With DM-ILD

**Figure 1:**
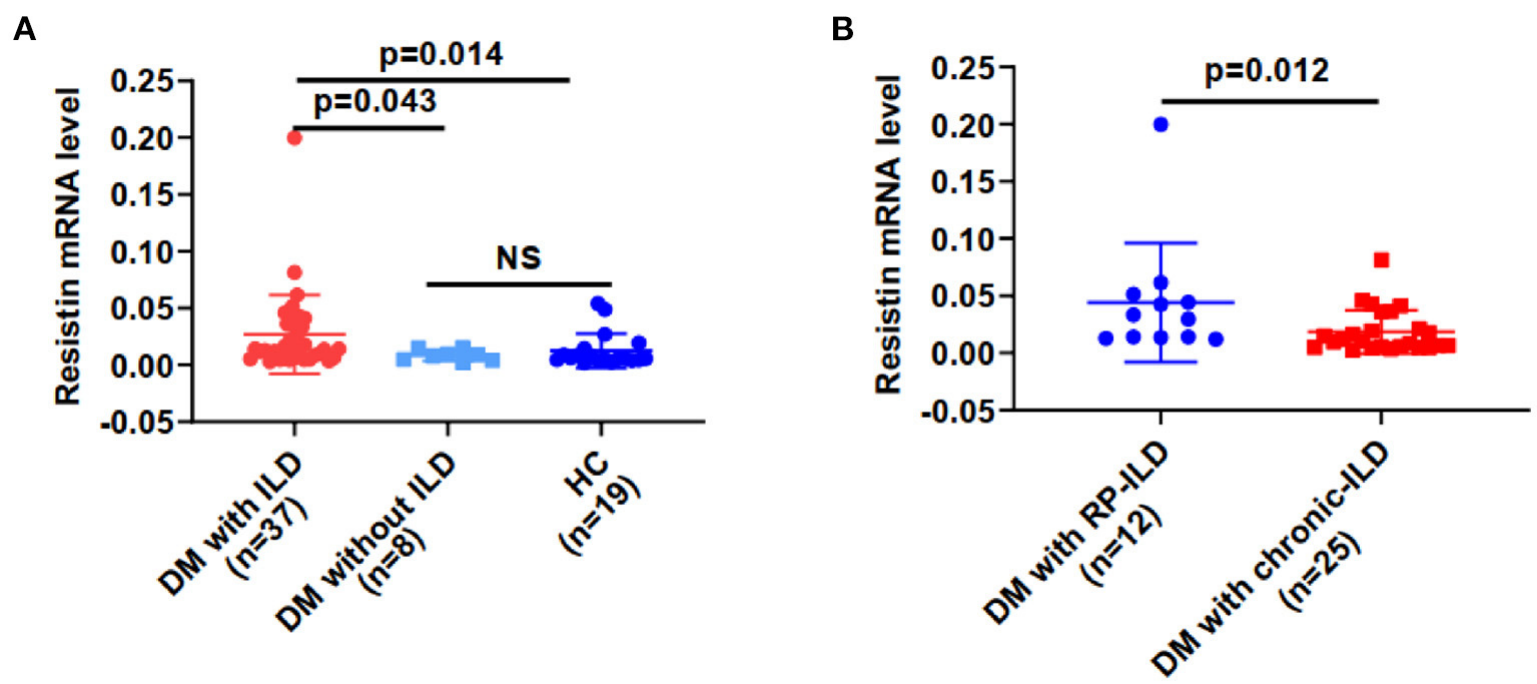
Resistin mRNA levels were higher from PBMCs in DM patients with ILD than in HCs. **(A)** Resistin mRNA levels in DM patients with ILD, without ILD, and in HCs. **(B)** Resistin mRNA levels in patients with DM and RP-ILD or chronic ILD. DM, dermatomyositis; HC, healthy control; ILD, interstitial lung disease; RP-ILD, rapidly progressive interstitial lung disease. The relative resistin expression levels in each group were determined *via* the 2^−Δ*Ct*^ method. The data shown are expressed as the mean ± SD.

Resistin mRNA levels differed significantly in PBMCs from DM patients with ILD, without ILD and HCs. The median resistin mRNA levels in DM-ILD were 0.014 (0.006–0.038), which was significantly higher than that in DM patients without ILD [0.008 (0.004–0.013)] and HCs [0.005 (0.004–0.014)] (*p* = 0.043, 0.014, respectively) (Figure 1A). No statistical difference was observed between DM patients without ILD and HCs. The mean age of the HCs was 49.79 years, and 13 (68.4%) of the HCs were women. DM-ILD and HCs had no significant differences in age (*p* = 0.31) or sex (*p* = 0.94). DM-ILD consist of two subgroups: patients with chronic ILD and patients with RP-ILD, and their resistin mRNA levels in PBMCs were compared (Figure 1B). Interestingly, the resistin mRNA levels (range) in patients with RP-ILD were significantly greater than those in patients with chronic ILD (0.031, 0.013–0.049 vs. 0.010, 0.005–0.028, respectively; *p* = 0.012).

### Correlations of Resistin mRNA Levels With Laboratory Data in DM-ILD

**Figure 2:**
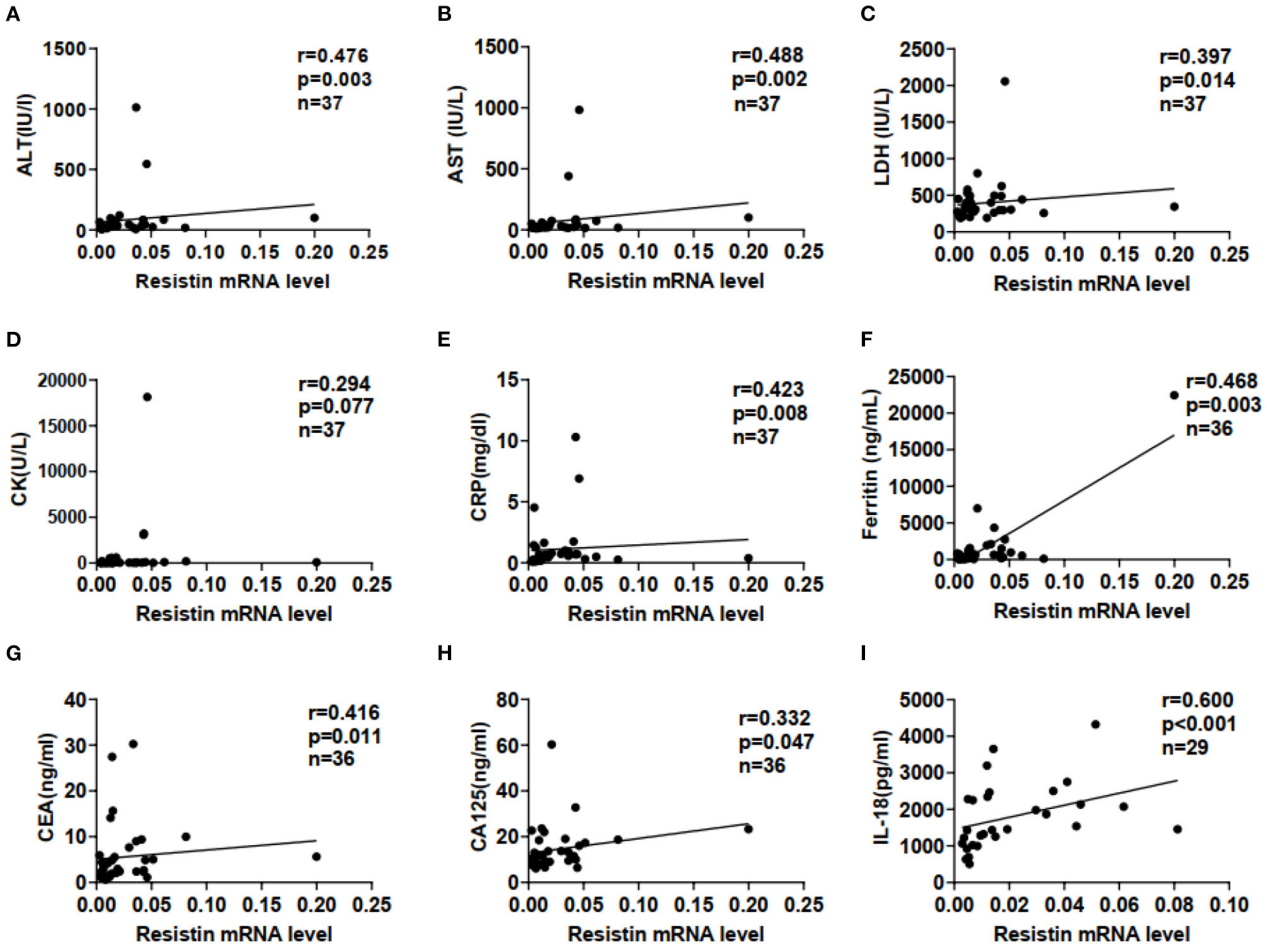
Elevated resistin mRNA levels in PBMCs were associated with laboratory data for patients with DM-ILD. Correlation of resistin mRNA levels in PBMCs with **(A)** alanine aminotransferase (ALT), **(B)** aspartate aminotransferase (AST), **(C)** lactate dehydrogenase (LDH), **(D)** creatine kinase (CK), **(E)** C-reactive protein (CRP), **(F)** ferritin, **(G)** carcinoembryonic antigen (CEA), **(H)** carbohydrate antigen (CA)125, and **(I)** interleukin-18 (IL-18) levels. Correlations were assessed using Spearman's correlation analyses.

We examined correlations between resistin mRNA levels in PBMCs and laboratory data from patients with DM-ILD. Resistin mRNA levels were significantly associated with the levels of alanine aminotransferase (ALT; r = 0.476, *p* = 0.003), aspartate aminotransferase (AST; r = 0.488, *p* = 0.002), lactate dehydrogenase (LDH; r = 0.397, *p* = 0.014), C-reactive protein (CRP; r = 0.423, *p* = 0.008), ferritin (r = 0.468, *p* = 0.003), carcinoembryonic antigen (CEA; r = 0.416, *p* = 0.011), and carbohydrate antigen (CA)125 (r = 0.332, *p* = 0.047), as shown in Figures 2A−2H. No significant correlations were found between resistin mRNA levels in PBMCs and creatinine kinase (Figure 2D). In addition, significant association was observed between resistin mRNA levels in PBMCs and serum IL-18 levels (r = 0.600, *p* < 0.001; Figure 2I).

### Elevated Resistin mRNA Levels in PBMCs Correlated With the Severity of Lung Involvement in DM-ILD

**Figure 3:**
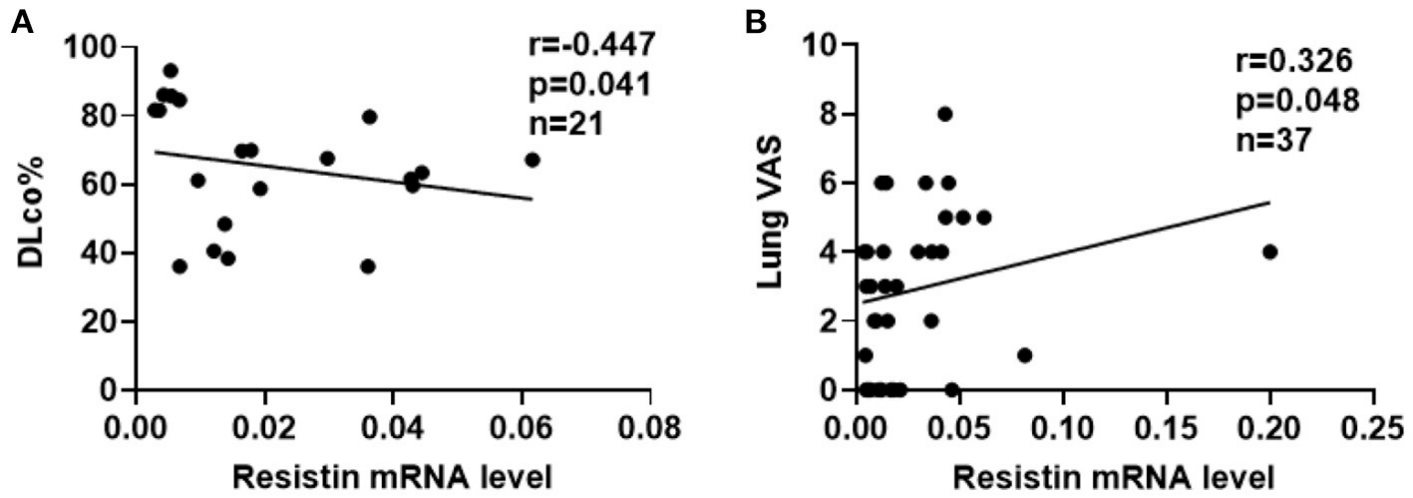
Elevated resistin mRNA levels in PBMCs correlated with the severity of lung involvement in DM-ILD. **(A)** Resistin mRNA levels were negatively associated with the DLco% in patients with DM-ILD. **(B)** Correlation between resistin mRNA levels and lung VAS values in DM-ILD. DLco, carbon monoxide diffusion capacity; DM-ILD, dermatomyositis-related interstitial lung disease; VAS, visual analog scale.

To explore the associations between resistin mRNA levels in PBMCs and the severity of lung involvement in DM-ILD, we analyzed correlation's between resistin mRNA levels and pulmonary function test (PFT) parameters. We found that the diffusing capacity of carbon monoxide (DLco)% negatively correlated with resistin mRNA levels (r = −0.447, *p* = 0.041) (Figure 3A). No significant associations were found between resistin mRNA levels in PBMCs and the forced vital capacity (FVC) % (data not shown). Furthermore, the lung VAS scores were evaluated when the blood samples were collected. We found that resistin mRNA levels were positively associated with lung VAS scores (r = 0.326, *p* = 0.048) (Figure 3B). The results revealed that DM-ILD had higher resistin mRNA levels with more severe pulmonary symptoms.

### Changes in Resistin mRNA Levels and Their Correlations With DM-ILD Activity

**Figure 4:**
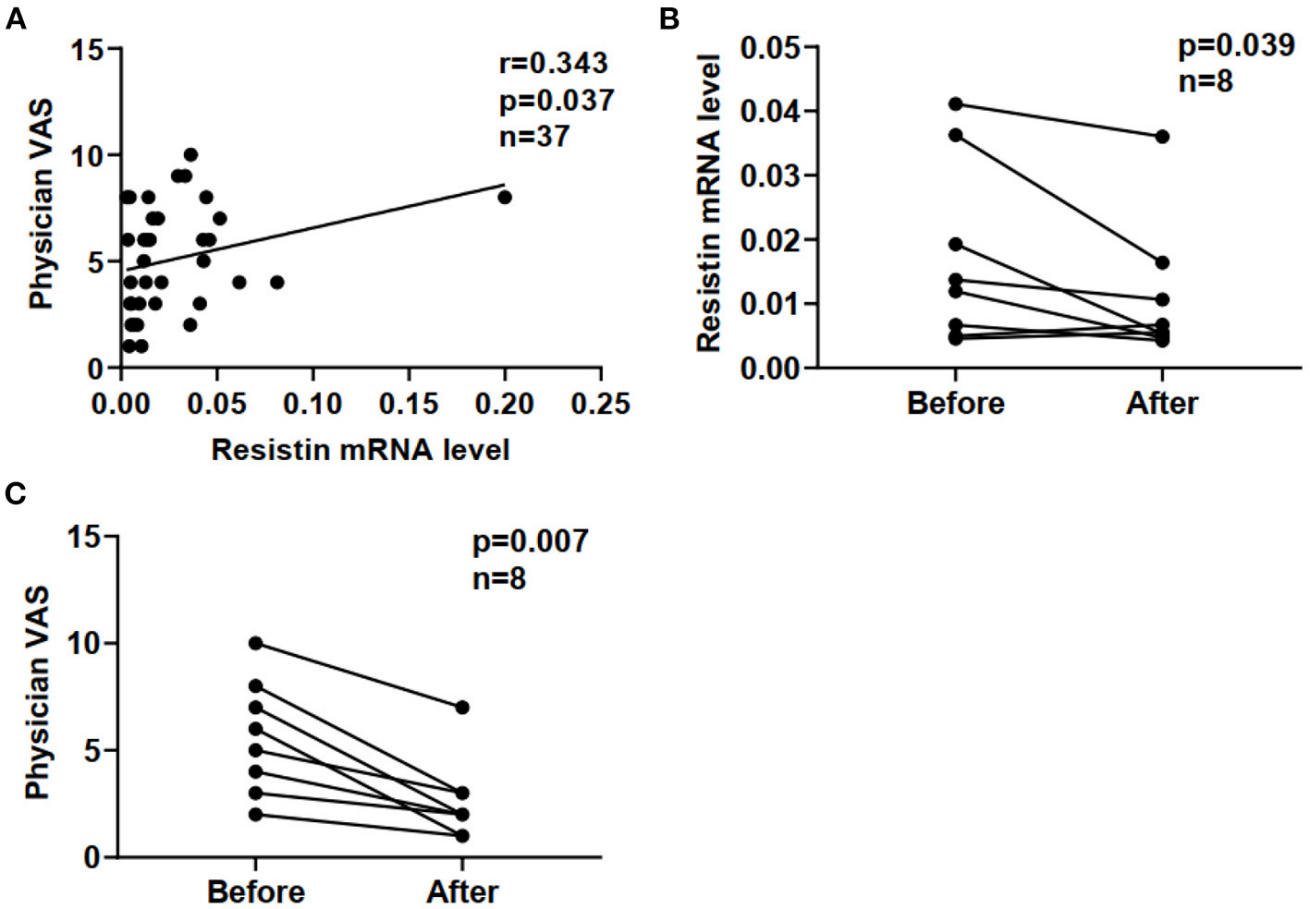
Correlation between resistin mRNA levels and disease activities in DM-ILD. **(A)** Correlation between resistin mRNA levels and physician VAS scores. **(B)** Resistin mRNA levels before and after treatment. **(C)** Physician VAS scores before and after treatment. Paired analysis was assessed by Wilcoxon's signed-rank test. DM, dermatomyositis; ILD, interstitial lung disease.

Furthermore, we compared resistin mRNA levels in PBMCs with disease activities in DM-ILD. In a cross-sectional study of 37 patients with DM-ILD, we found that resistin mRNA levels positively correlated with physician VAS scores (r = 0.343, *p* = 0.037) (Figure 4A). Clinical evaluations and blood tests were performed at each follow-up visit. Repeat blood samples were obtained from eight patients with DM-ILD (median pre-treatment to post-treatment interval; 12 [IQR: 3.5–16] months) before and after treatment. We tested the correlation between resistin mRNA levels and DM-ILD activities. Significantly decreased resistin mRNA levels in PBMCs were observed after immunosuppressive treatment (*p* = 0.039) (Figure 4B). The physician VAS scores also decreased after immunosuppressive treatment (*p* = 0.007) (Figure 4C).

### Tissue Expression of Resistin in Patients With DM-ILD

**Figure 5:**
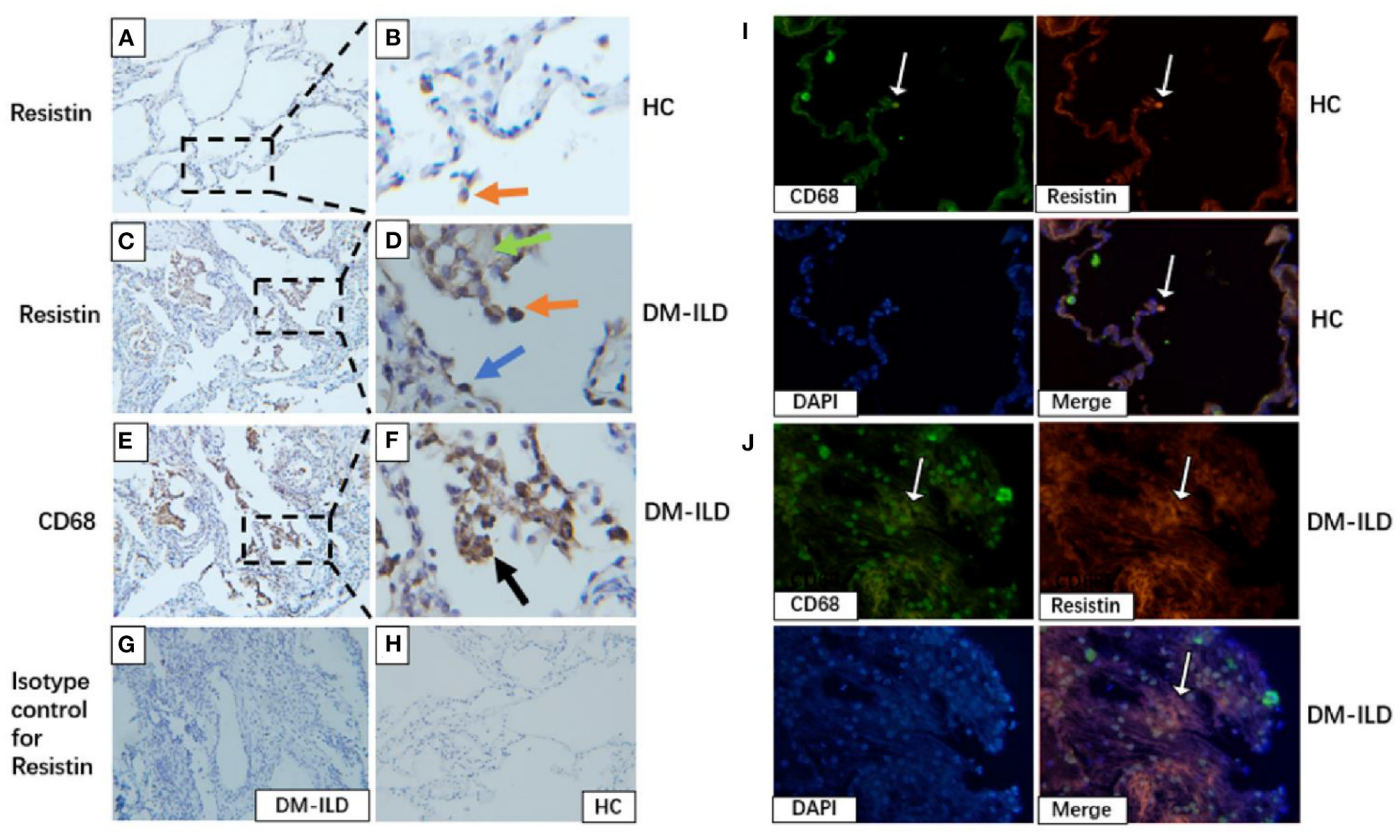
Elevated resistin expression in the lung tissue of patients with DM-ILD. **(A,B)** Lung of healthy control: A small number of resistin–positive macrophages (red arrowhead). Scale bar = 50 μm. **(C,D)** Lung of patients with DM-ILD: Resistin was strongly expressed in macrophages (red arrowhead), alveolar epithelial cells (blue arrowhead), and weak fibrotic lesions (green arrowhead). Scale bar = 50 μm. **(E,F)** Lung of patients with DM-ILD: Immunohistochemistry analysis on serial sections using an anti-CD68 antibody confirmed that resistin-positive cells were mostly macrophages (black arrowhead). Scale bar = 50 μm. **(G,H)** Isotype-matched control antibody for resistin in DM-ILD and HC. Scale bar = 50 μm. **(I)** Lung of healthy control: Two color immunofluorescence of resistin (red) with CD68+ macrophages (green), DAPI (blue). Scale bar = 50 μm. **(J)** Lung of patients with DM-ILD: Two color immunofluorescence of resistin (red) with CD68+ macrophages (green). Scale bar = 50 μm. DM, dermatomyositis; ILD, interstitial lung disease; HC, healthy control.

We investigated resistin expression in lung tissues obtained from patients with DM-ILD (*n* = 3) and HCs (*n* = 3) by performing immunohistochemical (IHC) staining. Lung sections from HCs showed a small number of resistin-positive macrophages (Figures 5A,B), whereas the lung sections from DM-ILD showed clear positive staining of resistin in macrophages, alveolar epithelial cells, and weak fibrotic lesions (Figures 5C,D). IHC staining for CD68 in DM-ILD confirmed that most resistin-expressing cells were macrophages (Figures 5E,F). IHC staining with an isotype-matched control antibody for resistin in DM-ILD and HC was also performed (Figures 5G,H). Furthermore, we performed immunofluorescence staining to confirm this as well. Two-color immunofluorescence was performed with resistin and CD68. CD68+ macrophages co-express resistin, which is rare in HC lung tissue (Figure 5I). However, in DM-ILD, a large number of CD68+ macrophages were seen co-expressing resistin (Figure 5J).

## Discussion

In this study, we revealed that resistin mRNA levels in PBMCs were obviously increased in patients with DM-ILD, especially in those with RP-ILD, and associated with the disease severity. We also found that resistin mRNA levels were associated with its severity in DM-ILD. Furthermore, resistin expression were more obvious in lung tissue specimens from patients with DM-ILD than in those from normal lungs. To our knowledge, this study is the first to determine the clinical significance of resistin in DM-ILD.

Previous studies have revealed that resistin levels were elevated in the synovial fluid of patients with RA and were positively associated with RA disease activity and joint damage (10). Similarly, compared with HCs or patients with osteoarthritis, serum resistin levels were increased in patients with RA and positively correlated with inflammatory markers such as ESR, CRP, and disease activity, which determined by measuring disease activity score 28 values. These studies indicated that resistin play a role in the regulation of inflammatory processes in RA (10, 24). Baker et al. revealed that serum resistin levels were increased in patients with SLE than in control subjects, and higher resistin levels were positively correlated with renal insufficiency, inflammatory markers, and disease damage (12). In another study, the serum and urine resistin levels of patients with lupus nephritis (LN) were elevated, which correlated with the relevant indicators of LN renal insufficiency and could be used as a potential marker of LN nephropathy (25). Filkova et al. observed higher serum resistin levels in patients with IIM, which strongly associated with CRP levels and the overall disease activity. In addition, resistin levels were significantly associated with overall disease activities and muscle enzyme levels in patients with DM (15). Similarly, serum resistin levels were elevated in young adult female patients with DM and correlated with age and disease activity (26). Another study showed that serum resistin levels were increased in patients with antisynthetase syndrome (26, 27). In our study, we found that resistin mRNA levels in PBMCs were higher in DM-ILD than in DM patients without ILD and normal controls. We observed correlations between resistin levels and disease activity in DM-ILD, which was consistent with the above-mentioned studies. Resistin mRNA levels varied with disease activity, and trends between resistin levels and PGA VAS scores in some patients were also shown. These results suggest that resistin levels parallel disease severity, at least in some patients with DM-ILD. Therefore, resistin levels in patients with DM-ILD may reflect overall disease activity.

Resistin is a pro-inflammatory cytokine that plays an important role in the development of inflammation by regulating the production of various cytokines and chemokines (16, 28). Previous studies have revealed that resistin mediates the secretion of IL-1, IL-6, and TNF-α in human monocytes through the nuclear factor-kappa B signaling pathway (16, 28). These pro-inflammatory cytokines can strongly induce resistin expression in monocytes/macrophages, triggering a positive feedback loop for self-injury (16). Filkova et al. observed that resistin induced the expression of several proinflammatory mediators, such as IL-1, IL-6, monocyte chemoattractant protein-1, and TNFα, in PBMCs from patients with IIM (15). These pro-inflammatory cytokines are involved in mediating the inflammatory pathogenesis of DM-ILD (29). Resistin is primarily expressed by macrophages (9). Macrophage activation also participates in the pathogenesis of DM-ILD (30). Previous results have shown that serum CRP, ferritin, IL-18, and LDH levels are related to DM-ILD, confirming this view (3, 29). During ILD progression, macrophage activation can promote hepatocyte injury, resulting in increased ALT and AST levels (31). Previous data also showed that serum CEA and CA125 levels were closely related to DM-ILD (32). In our study, resistin mRNA levels positively correlated with the above-mentioned ILD-related inflammatory markers and cytokines. Therefore, resistin may be involved in ILD pathogenesis in patients with DM.

Data from several *in vivo* and *in vitro* studies suggest that resistin is involved in the pathogenesis of fibrotic conditions. First, the murine homolog, murine resistin-like molecule alpha (mRELMα) was significantly upregulated in a rat model of bleomycin-induced pulmonary fibrosis, which stimulated alpha-smooth muscle actin and collagen I production in fibroblasts (33). mRELMα exhibits a crucial profibrotic effect in experiments in mRELMα knockout mice and adenoviral mRELMα overexpressing rats (34). Pulmonary fibrosis due to accumulation of asbestos and silica is correlated with high serum resistin levels (35, 36). These data revealed that resistin is involved in the immune response to fiber- or particle-induced fibrotic disease and may serve as a biomarker (35, 36). In a recent study, plasma and sputum resistin levels were elevated in patients with cystic fibrosis-related lung disease and were associated with impaired lung function (17). Elevated serum resistin levels were also associated with organ involvement in SSc, including ILD (11). Our current findings also showed that resistin was associated with ILD. First, we found that resistin was elevated in PBMCs and lung tissues from patients with DM-ILD. Immunostaining of ILD specimens showed that resistin was expressed in both inflammatory cells and interstitial fibrosis tissue, suggesting that resistin may participates in inflammation and fibrosis in DM-ILD. Immunofluorescence staining confirmed CD68+ macrophages co-express resistin. Second, significantly higher resistin mRNA levels were detected in the patients with DM and RP-ILD. Furthermore, resistin mRNA levels positively correlated with ILD-related inflammatory markers and cytokines. Therefore, resistin may be involved in ILD pathogenesis in DM, and higher inflammation and fibrosis in RP-ILD may lead to higher resistin mRNA levels than in chronic ILD. Third, we found that resistin mRNA levels in DM-ILD correlated with DLco% and lung VAS scores. These metrics help to quantify the severity of ILD. Therefore, resistin mRNA levels are indicative of DM-ILD severity and disease activity (37).

This study had some limitations. First, the small sample size and single-center study design may have led to selection bias, and larger population-based multicenter studies are needed to confirm our preliminary results. Second, due to the retrospective design, some patients did not undergo laboratory tests for ferritin and lung function, so statistical bias could not be avoided. Third, the limited number of available lung-biopsy samples affected the statistics of the immunohistochemical results.

In conclusion, the current findings preliminarily show that resistin levels were elevated in PBMCs from patients with DM-ILD, especially those with RP-ILD. Furthermore, we demonstrated that resistin mRNA levels correlated with various clinical variables, including the DLco %. Therefore, resistin mRNA levels may serve as a marker of disease activity in DM-ILD. A prospective multicenter study is needed to elucidate the role of resistin in DM-ILD pathogenesis.

## Data Availability Statement

The data presented in the study are available from the corresponding authors upon request.

## Ethics Statement

The studies involving human participants were reviewed and approved by the Ethics Committee of China–Japan Friendship Hospital. The patients/participants provided their written informed consent to participate in this study. Written informed consent was obtained from the individual(s) for the publication of any potentially identifiable images or data included in this article.

## Author Contributions

LY, YX, PZ, HY, and SL participated in collecting the data and samples. YZ, FC, and QP participated in experimental design. GW and XS supervised the manuscript. LY wrote the manuscript. All authors contributed to the article and approved the submitted manuscript.

## Funding

This work was supported by the Elite Medical Professionals project of China-Japan Friendship Hospital (No. ZRJY2021-GG14), the Youth Program of the National Natural Science Foundation of China (Grant Numbers 81401363 and 81601367), National Natural Science Foundation of China (Grant Number 81971531), Capital Health Research and Development of Special Programs (Grant Number 2014–4-4062), and the Fundamental Research Funds for the Central Universities (Grant Number 3332020074).

## Conflict of Interest

The authors declare that the research was conducted in the absence of any commercial or financial relationships that could be construed as a potential conflict of interest. The handling editor JZ declared a shared secondary affiliation with the authors LY, YX, SL, and GW at the time of review.

## Publisher's Note

All claims expressed in this article are solely those of the authors and do not necessarily represent those of their affiliated organizations, or those of the publisher, the editors and the reviewers. Any product that may be evaluated in this article, or claim that may be made by its manufacturer, is not guaranteed or endorsed by the publisher.
